# Colistin Resistance Mechanisms in Human *Salmonella* *enterica* Strains Isolated by the National Surveillance Enter-Net Italia (2016–2018)

**DOI:** 10.3390/antibiotics11010102

**Published:** 2022-01-13

**Authors:** Daniela Fortini, Slawomir Owczarek, Anna Maria Dionisi, Claudia Lucarelli, Sergio Arena, Alessandra Carattoli, Enter-Net Italia Colistin Resistance Study Group, Laura Villa, Aurora García-Fernández

**Affiliations:** 1 Department of Infectious Diseases, Istituto Superiore di Sanità, 00161 Rome, Italy; daniela.fortini@iss.it (D.F.); slawomir.owczarek@iss.it (S.O.); annamaria.dionisi@iss.it (A.M.D.); claudia.lucarelli@iss.it (C.L.); sergio.arena@iss.it (S.A.); laura.villa@iss.it (L.V.); 2 Department of Molecular Medicine, Sapienza University of Rome, 00161 Rome, Italy; alessandra.carattoli@uniroma1.it

**Keywords:** *Salmonella*, Enteritidis, Typhimurium, colistin, *mcr*, antibiotic resistance, zoonosis, IncX4, IncHI2

## Abstract

Background: A collection of human-epidemiologically unrelated *S. enterica* strains collected over a 3-year period (2016 to 2018) in Italy by the national surveillance Enter-Net Italia was analysed. Methods: Antimicrobial susceptibility tests, including the determination of minimal inhibitory concentrations (MICs) for colistin, were performed. Colistin resistant strains were analysed by PCR to detect mobile colistin resistance (*mcr*) genes. In *mcr*-negative *S. enterica* serovar Enteritidis strains, chromosomal mutations potentially involved in colistin resistance were identified by a genomic approach. Results: The prevalence of colistin-resistant *S. enterica* strains was 7.7%, the majority (87.5%) were *S*. Enteritidis. *mcr* genes were identified only in one strain, a *S*. Typhimurium monophasic variant, positive for both *mcr-1.1* and *mcr-5.1* genes in an IncHI2 ST4 plasmid. Several chromosomal mutations were identified in the colistin-resistant *mcr*-negative *S*. Enteritidis strains in proteins involved in lipopolysaccharide and outer membrane synthesis and modification (RfbN, LolB, ZraR) and in a component of a multidrug efflux pump (MdsC). These mutated proteins were defined as possible candidates for colistin resistance in *mcr*-negative *S*. Enteritidis of our collection. Conclusions: The colistin national surveillance in *Salmonella* spp. in humans, implemented with genomic-based surveillance, permitted to monitor colistin resistance, determining the prevalence of *mcr* determinants and the study of new candidate mechanisms for colistin resistance.

## 1. Introduction

The spread of multidrug-resistant (MDR) Gram-negative bacteria (GNB) yielding nosocomial infections is a growing problem worldwide. Colistin (COL), also called polymyxin E, and polymyxin B have been considered two of the last-resort treatments for such infections. Although the small use in human medicine in the past due to neurotoxicity and nephrotoxicity, COL has been widely used in veterinary medicine to promote animal growth in the livestock and seafood industry [1]. The recent rise in COL consumption in human medicine due to the emergence of MDR Enterobacterales and the overuse and/or misuse of COL among animals have led to the global emergence of COL-resistant (COL-R) pathogens [2].

In 2016, the WHO classified polymyxins into the group of critically important antimicrobials (CIA) with the highest priority (HPCIA) for human medicine [3]. Moreover, the World Organization for Animal Health (OIE) included this class of antimicrobials, in their list of veterinary antimicrobial agents, into the category of high importance [4]. COL resistance represents a critical One Health dimension of antimicrobial resistance. A One Health approach to combat antibiotic resistance in animal and human medicine and to prevent transmission of zoonotic diseases was stated in 2017. The zoonotic agent *Salmonella enterica* represented an important reservoir of COL resistance through its transmission between animals and humans, to humans/animals via contaminated food, and through the environment. *S. enterica* could also play a significant role in disseminating *mcr* genes to other pathogens with clinical relevance.

COL acts mainly on the GNB cell wall altering its structure through electrostatic interactions with lipopolysaccharides (LPS); it disrupts the outer membrane causing an osmotic imbalance that leads to cell death [5]. In recent years, many studies have indicated that the prevalence of COL resistance has increased rapidly among Enterobacterales [6]. The mechanisms underlying polymyxins resistance in GNB are complex and not completely understood.

Several species are intrinsically resistant to this antimicrobial. These include *Serratia marcescens*, *Proteus* spp., *Providencia* spp., *Morganella morganii*, *Vibrio cholerae*, *Brucella*, *Campylobacter* spp., *Legionella* spp., *Chromobacterium*, *Neisseria* spp., *Edwardsiella* spp., some *Aeromonas* species and *Burkholderia cepacian* [7,8].

In *Klebsiella*, *Escherichia coli*, *Shigella*, *Citrobacter*, *Proteus*, *Enterobacter* and *Salmonella*, the most common LPS modifications associated with increased MIC to COL are (i) the cationic substitution of the phosphate groups by 4-amino-4-deoxy-l-arabinose (l-Ara4N), which decreases the negative charge of lipid A; (ii) the phosphoethanolamine (PEtN) modification causing a net positive charge of the modified LPS that reduces its interaction with polymyxins, increasing resistance to COL [7,9]. Furthermore, additional genetic polymorphisms in genes of the two-component system (TCS) (PhoPQ, PmrAB, ParRS, ColRS, CprRS) and their regulators (MgrB, PmrD, PmrC, PmrE and the PmrHFIJKLM operon) involved in lipid A biosynthesis have also been identified as responsible for COL resistance in GNB [7,10,11,12,13]. Mutations in genes and operons essential for the lipid A formation, such as *lpxA*, *lpxC*, *lpxD*, have also been described [14,15]. *S. enterica* polymyxin-resistance is modulated mainly by substituting the acyl chains, the phosphate groups on the lipid A moiety of LPS and alterations into membrane fluidity/permeability [7,16]. It has also been described that the O-antigen epitope in *Salmonella* group D, to which *S*. *enterica* serovar Enteritidis belongs, governs the levels of COL susceptibility [17]. The O-antigens of *Salmonella* group D differ from group B since they have tyvelose in place of abequose as the side-branch sugar. Increased COL susceptibility in *Salmonella* group D was also due to a frameshift mutation in the *rfc* gene, which encodes the O-antigen polymerase [17]. Other alterations, such as deacylation of lipid A by PagL [11] and activation of transcription of genes involved in adaptation and survival of the bacterial cells by RpoN [16], could also lead to COL resistance in *S. enterica*. The efflux is also likely involved in COL resistance, often resulting from combined resistance mechanisms of defects in outer membrane proteins and structural modification of the LPS [11,18]. In *S. enterica,* a periplasmatic protein (YdeI), regulated by the PhoPQ and PmrAB TCSs, interacts with the OmpD porin increasing resistance to COL [18].

Acquired COL resistance by horizontal transfer of the *mcr-1* gene was first described in *E. coli* [19]. Currently, ninety-eight *mcr* alleles distributed in the ten gene variants (from *mcr-1* to *mcr-10*) have been identified worldwide (https://www.ncbi.nlm.nih.gov/pathogens/refgene/#, accessed on 3 June 2021), and six of them (*mcr-1,-2,-3,-4,-5,-9*) were reported in *S. enterica* [20,21,22,23,24,25]. The *mcr* genes encode a PEtN transferase that modifies cell membrane lipid A head groups with a PEtN residue, reducing affinity to COL [20]. *mcr* genes have been detected in animal and human isolates located on a wide range of conjugative plasmids, mainly in IncI2, IncHI2, IncX4 and less frequently in the IncF type [26].

This study aims to investigate the prevalence of COL resistance in a *S. enterica* collection isolated from humans in Italy during a 3-year period (2016 to 2018). Mechanisms responsible for COL resistance were investigated. The plasmid-mediated mechanisms and the potentially chromosomal mutations responsible for COL resistance were examined by whole-genome sequencing (WGS) in some isolates of this collection. The prevalence of COL resistance in our collection was low. The *mcr*-mediated resistance genes were identified in only one strain, whose plasmid was sequenced entirely. *S.* Enteritidis, the most frequent COL-R serogroup in our 2016–2018 collection, was not positive for known *mcr*-genes. However, some novel mutations in genes potentially involved in lipid A formation and modification and in efflux pumps were identified by WGS in some isolates of this collection.

## 2. Results

### 2.1. Antimicrobial Susceptibility and Mcr-Gene Testing

A total of 289/313 (92.3%) *S. enterica* strains showed COL-S MIC ≤ 2 mg/L, and 24 (7.7%) were COL-R (MIC > 2 mg/L), showing MIC = 4 mg/L.

Only three serotypes were COL-R, 21 (87.5%) isolates were *S.* Enteritidis, 2 (8.3%) *S.* Typhimurium monophasic variant and 1 (4.2%) *S.* Typhimurium (Supplementary Table S1). PCR for *mcr-1* to *mcr-10* genes in COL-R strains detected only one positive strain (1/24 = 4.2%), a *S*. Typhimurium monophasic variant isolated in 2016 (77/84/18) carrying both *mcr-1* and *mcr-5* genes. No *Salmonella* isolates resulted positive for *mcr-2* to *mcr-4* and for *mcr-6* to *mcr-10* genes. Among the COL-R *mcr*-negative strains collected in 2016, one was *S*. Typhimurium monophasic variant, one was *S*. Typhimurium and six were *S*. Enteritidis. In 2017 and 2018, eleven and four COL-R *mcr*-negative strains, respectively, were identified, all belonging to the *S.* Enteritidis serovar.

Antimicrobial susceptibility to other antimicrobials was tested on 212 (66.7%) randomly selected *S*. *enterica* strains of this study (Supplementary Table S1). 37.2% of the tested strains were MDR, defined as non-susceptible to at least one agent in three or more antimicrobial categories [27]. The most frequent MDR pattern was ASSuT (11.8%), mainly found in *S*. Typhimurium monophasic variant. Extended-spectrum beta-lactamase production was found in 4.2% of the strains, prevalently in *S*. Infantis and *S*. Typhimurium. Quinolone resistance was present in 13.2% of the strains (Supplementary Table S1).

### 2.2. Genomic Characterisation of Isolates

Among the 21 COL-R *S.* Enteritidis, three strains, one for each year of surveillance, were selected for WGS. Strains 58/10/16 (the last strain identified in 2016) and 83/46/17 were isolated at one year of distance in late 2016 and 2017, respectively. Strain 45/7/18 was the last COL-R strain isolated in our collection (May 2018, Supplementary Table S1). Among the susceptible strains, two COL-S *S.* Enteritidis strains, 56/1/16 and 4/23/16, with COL MIC = 2.0 mg/L and MIC = 1.0 mg/L, respectively, were selected for WGS. WGS was performed on the COL-R *S*. Typhimurium monophasic variant 77/84/18 strain, positive for *mcr-1* + *mcr-5* genes, and on the COL-R *mcr*-positive *S.* Enteritidis strain, isolated in 2009, for comparison (Table 1).

Genomic analysis revealed that GC content was 51.9–52.1%, and the N50 of 203,934–406,120 bp and de novo assembly yielded 49–87 contigs (≥200 bp) for each isolate with the total length ranging from 4,643,868 to 5,130,079 bp. Overall, 4646–5266 genes and 4454–5266 protein-encoding sequences were annotated from each draft genome (data not shown).

The serotype of each sequenced *Salmonella* spp. strain was confirmed by SerotypeFinder 2.0. The MLST obtained through the genome sequence was ST34 for the *S*. Typhimurium monophasic variant. Two out of four COL-R *mcr*-negative *S.* Enteritidis strains were ST11, a worldwide distributed clone [28]. The others were ST3645 and ST3233, respectively. The COL-S *S*. Enteritidis strains were all ST11 (Table 1). The COL-R *mcr*-positive *S*. Enteritidis strain from 2009 was also ST11.

Whole-genome single-nucleotide polymorphism (SNP)-based phylogenetic analysis revealed that COL-S strains were localised in the same branch of COL-R strains, indicating high levels of homology among these isolates (Supplementary Figure S1). SNP analysis revealed that the five *S.* Enteritidis genomes, identified in the 2016–2018 period, were related in the average of 36–97 SNPs, independently by the COL-S and COL-R phenotype. Furthermore, they were highly distant from the COL-R *mcr*-positive *S.* Enteritidis strain 61/4/09 identified in 2009 (479–494 SNPs; Supplementary Figure S1). The COL-R 83/46/17 and 45/7/18 strains were highly related to COL-S 56/1/16 and 4/23/16 strains, respectively (36 and 59 SNPs; Supplementary Figure S1). 

cgMLST was determined for all *S*. Enteritidis strains subjected to WGS. This analysis revealed that COL-S differed by 17–44 allele distance (AD) from COL-R strains. The samples pair COL-R/COL-S with lower AD was COL-S 56/1/16 with COL-R 83/46/17 (17 AD), and the samples pair with higher AD was COL-S 4/23/16 with COL-R 58/10/16 (44 AD) (Supplementary Table S3).

The five COL-R genomes were screened in silico by AMRFinderPlus Tool and ResFinder, selecting a threshold of 60% of identity (Table 1). This analysis confirmed the presence of *mcr*-genes and other multiple acquired antibiotic resistance genes in the *S*. Typhimurium monophasic variant 77/84/18 and in the *S*. Enteritidis 61/4/09 strains. Interestingly, two *mcr*-genes, namely *mcr-1.1* and *mcr-5.1* gene variants, were both identified in the 77/84/18 strain. No other known plasmid or chromosome-located COL-resistance mechanisms were identified in the COL-R *mcr*-negative *S*. Enteritidis strains (Table 1). The AMRFinderPlus Tool revealed the presence of the metal resistance genes *golTS* in all the strains (Table 1).

### 2.3. Chromosomal Mutations Study

Genomic analysis comparing COL-R *mcr*-negative *S*. Enteritidis strains (58/10/16, 83/46/17 and 45/7/18) with the COL-S *S*. Enteritidis 4/23/16 and 56/1/16 strains revealed from 1 to 10 deleterious mutated proteins, depending on the compared strains (Supplementary Table S2). These proteins were not mutated in the two sequenced COL-S *S*. Enteritidis strains. Only deleterious mutations, using the Provean prediction tool, were considered. Among deleterious mutated proteins, six belonged to pathways already described to be correlated with COL MIC increment in *S. enterica* or other GNB species (Table 2). Of the six mutated candidate proteins, four were identified in 58/10/16 strain and two in 45/7/18 strain; any mutated protein potentially associated with known mechanisms for COL resistance was found in the COL-R *mcr*-negative *S*. Enteritidis strain 83/46/17.

In COL-R *mcr*-negative *S*. Enteritidis 45/7/18 strain, the two proteins with deleterious mutations potentially associated with COL-R were (i) the D107V mutation in the O-antigen biosynthesis rhamnosyltransferase protein RfbN (also called WbaN); (ii) the R26L mutation in the transcriptional regulatory protein ZraR (Table 2).

The COL-R *mcr*-negative *S*. Enteritidis 58/10/16 strain showed four deleterious mutations (i) the R298C in MdsC, an outer membrane lipoprotein that is part of the MdsAB-MdsC tripartite efflux pump; (ii) the P107L in PilN, the Type IV pilus biogenesis protein; (iii) the R127L in YdeI, a family stress tolerance OB-fold protein; (iv) the S91R in LolB, an outer membrane lipoprotein receptor (Table 2).

Blastp analysis was performed against the RefSeq NCBI, non-redundant protein database, restricting the search to the subset *S. enterica*. The MdsC, PilN, YdeI, LolB, ZraR and RfbN wild type protein sequences, as identified in the two COL-S *S*. Enteritidis strains, returned a minimum of 95 matches at 100% amino acid identity (data not shown).

Differently, MdsC, PilN, YdeI and LolB mutated proteins returned only four matches with 100% amino acid identity, demonstrating that these mutations were rarely present in *Salmonella* genomes in GenBank. For ZraR, there were no proteins with 100% amino acid identity in GenBank. Blastp analysis of the D107V-mutated RfbN identified four *S. enterica* proteins showing a different aminoacidic residue at position D107 (Supplementary Table S3).

The genes encoding the four PilN, YdeI, LolB and ZraR proteins were identified within the 3002 loci of cgMLST, as STMMW_34811, STMMW_15151, STMMW_17701 and STMMW_41271 loci, respectively, while genes encoding the MdsC and RfbN proteins were not included in the cgMLST database (Supplementary Table S3).

Different cgMLST alleles were identified for the PilN, YdeI, LolB and ZraR genes in the COL-S and COL-R *S*. Enteritidis genomes, respectively. The alleles of mutated proteins, respectively identified in 58/10/16 and 45/7/18 isolates, were rarely represented in the pubMLST site [29], being present in less than 46 records. In contrast, the alleles corresponding to wild type proteins from the COL-S genomes were detected in almost 4000 records in the pubMLST site (Supplementary Table S3).

### 2.4. Characterisation of the Mcr-Positive Plasmids

PlasmidFinder and the pMLST/FAB formula revealed the presence of the IncF multi-replicon virulence plasmid of *Salmonella* (FIIs) with the FAB formula [S1:A-:B22] in all *S*. Enteritidis strains. *S*. Typhimurium monophasic variant 77/84/18 did not present any IncF multi-replicon plasmid and was positive for Col, Q1 and HI2 replicons. The IncHI2 plasmid was classified as ST4 by the plasmid double locus sequence typing (pDLST). The COL-R *mcr*-positive *S*. Enteritidis 61/4/09 strain showed co-resident IncF [S1:A-:B22], IncX4 and IncN plasmids (Table 1).

The genomic analysis localised the *mcr1.1* gene in the IncX4 plasmid in the COL-R *S*. Enteritidis 61/4/09 strain. This plasmid, named p61/4/09-IncX4, was 33,360 bp in length with GC content of 52.1%, presenting a typical IncX4 plasmid backbone, with genes involved in plasmid replication, maintenance and transfer. This plasmid was closely related to another IncX4 plasmid carrying *mcr-1*, pMCR1.2-IT (KX236309), isolated in Italy, with an identity at a nucleotide sequence level of 99.99%. The *mcr-1.1* gene was surrounded upstream by a hypothetical protein and downstream by the *pap2* gene, coding for a transmembrane protein, lacking both copies of IS*Apl1* and the putative inverted repeat sequences of the ancestral Tn*6330* (IS*Apl1*-*mcr-1*-*pap2*-IS*Apl1*) as previously described [30,31,32,33].

**Figure 1:**
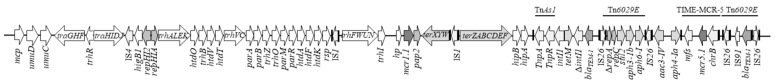
Major structural features of p77/84/18-IncHI2 (MZ666126). Predicted coding sequences are indicated in shades of grey arrows and oriented in the direction of transcription of each respective gene.

In the COL-R *S*. Typhimurium monophasic variant 77/84/18, the *mcr-1.1* and the *mcr-5.1* genes were colocalised in an IncHI2 plasmid containing IncQ1 Δ-*repA* and *repC* genes (Figure 1).

The IncQ1 deleted replicon was previously described and is likely unfunctional being trapped by mobilisation of the *aph(3″)-Ib, aph(6)-Id, sul2* genes within the Tn*6029* transposon [34]. The p77/84/18-IncHI2 ST4 plasmid was 227,392 bp in length with GC content of 50.3%. It showed 99.87% nucleotide identity and 89% coverage with pTZ41_1P_HI2 plasmid (MT604108). The plasmid backbone presented the IncHI2 transfer (*trh*), replication (*repHI2*) and partitioning (*par*) genes. The commonly IncHI2-associated tellurium-resistance operon (*terZABCDEF*) was also present (Figure 1). The *mcr-5.1* gene was in a structure named Tn*3*-derived inverted-repeat miniature element (TIME)-MCR-5 (Figure 1), bracketed by two inverted repeats (IR148 and IRL) and 5-bp direct repeats (DRs). A transposase-mediated acquisition has been suggested even if, as previously described, no transposase gene is present [32,35]. The TIME-MCR-5 region was inserted in a resistance region encoding *aac3-IV* and *aph4-Ia* genes, flanked by two direct copies of IS*26* (Figure 1). The plasmid had a complex antibiotic resistance locus (CRL), carrying a set of antibiotic resistance genes, four IS*26* copies (Figure 1). The Tn*6029*E transposon showed 99% identity with chromosomal islands in 105/7/03 *S*. Typhimurium monophasic variant and BD1380 *S*. Typhi strains [36,37]. This CRL of 11,400 bp also included a Tn*As1* chimeric transposon containing transposase and resolvase genes, with IRs and DRs, and unrelated genes associated with Tn*1722* [38]. Upstream the resolvase Tn*As1*, *tet(M)* gene was inserted, flanked by two integrase genes as described in the pCFS3292-1 IncHI2 plasmid (NZ_CP026936) (Figure 1).

## 3. Discussion

In Europe, COL resistance rates for *Salmonella* spp. and *E. coli* are generally low in humans. Only one country (the Netherlands), out of the seven countries reporting COL susceptibility data, presented a resistance rate higher than 15% for *Salmonella* [39]. Several studies in Europe revealed the increase of COL-R *Salmonella* strains in the veterinary field [40,41]. The European Union summary report 2019 reported COL resistance at overall low levels among isolates from turkeys, broilers, calves and laying hens (1.5%, 1.8%, 3.1% and 8.1%, respectively). From the monitoring of poultry in 2018, 89.6% of *S.* Enteritidis isolates were COL-R in laying hens [42]. However, few studies have been carried out in isolates from humans. Our study revealed that the COL resistance in *S. enterica* from humans in Italy is relatively low (7.7%). A previous regional Italian study, performed in the 2012–2015 period, demonstrated a prevalence of COL resistance of about 6.6% [43], so the COL resistance in *S. enterica* in our country is flat or slightly increasing.

The emergence of *mcr* genes has triggered concerns worldwide, and the prevalence of *mcr* genes in bacteria from humans, animals and food was recently investigated. *mcr* genes were reported from 47 countries across six continents, and the overall average prevalence was 4.7% (0.1–9.3%) [44]. The estimated prevalence of these genes in animal isolates suggests a role in the foodborne transmission of COL resistance. It is crucial to monitor COL resistance in humans’ zoonotic foodborne pathogens such as *Salmonella*.

This study identified only one *mcr*-positive *S*. Typhimurium monophasic variant isolated from the surveillance collection 2016–2018, indicating a low prevalence of *mcr* in humans in Italy. *S*. Typhimurium monophasic variant assigned to the ST34 is frequently observed in humans and animals [25]. The association of COL resistance genes (*mcr-1, mcr-3* and *mcr-5*) to MDR *S.* Typhimurium monophasic variant ST34 is globally well known [25].

Both *mcr-1.1* and *mcr-5.1* genes were observed in an IncHI2 ST4 plasmid in the MDR epidemic, ST34 *S*. Typhimurium monophasic variant. The *mcr-1.1 + mcr-5.1* p77/84/18-IncHI2 ST4 plasmid in this strain showed 99.87% nucleotide identity and 89% coverage with pTZ41_1P_HI2 plasmid, harboured by an *mcr*-negative commensal *E. coli* strain from a pig source in Australia [45]. The *mcr-1* gene was previously described in 11 different plasmid types: IncX3, IncX4, IncX3-X4 hybrid, IncHI1, IncHI2, IncP, IncI2, IncF, IncFII, IncI2–IncFIB hybrid and IncY [25,31,33,46,47,48]. The *mcr-5* gene has been described in IncX1, ColE-like and untypable plasmids [25,49]. The high *mcr-5* mobility through independent acquisitions of *mcr-5*-harbouring plasmids by individual strains, successful integration in the chromosome and adaptation to different genomic environments have been previously demonstrated [25]. A collection of *S*. Typhimurium ST34, isolated from pigs and meat, presented IncX1, ColE and untypable plasmids in Germany, harbouring the *mcr-5* gene on Tn*6452* (Tn*3* family transposon) or putative mobile insertion cassettes [25]. The high variation of *mcr-5*-carrying plasmids (ColE, IncX1 and untypeable plasmids), and the presence of imperfect Tn*3*-like inverted repeats and target site duplications usually generated during the insertion of Tn*3* transposons, suggest an independent acquisition of *mcr-5*-harboring plasmids by individual strains by successful integration and adaptation to different genomic environments [25]. In our isolate, the acquisition of *mcr-5.1* gene in IncHI2 plasmid, already known to present *mcr-1.1* gene variant, suggested independent acquisition events of mobile COL resistance genes by IncHI2 plasmid. The presence of *mcr-1.1 + mcr-5.1* may confer special advantage under COL selective pressure to the COL-R *S*. Typhimurium monophasic variant 77/84/18, also in case one of the *mcr* genes is lost.

The Tn*As1* transposase gene in the IncHI2 plasmid could be responsible for mobilising the *mcr-5.1* [35]. IS*26* plays a crucial role in disseminating antibiotic resistance genes in GNB and Tn*As1* chimeric transposon. The IS*26* increases the frequency of additional insertion events, forming regions containing more than one antibiotic resistance gene, as previously described [50]. This latter resistance region has been acquired by transposition mechanism, as suggested by the presence of two copies of DRs, in a Tn*6029E* variant transposon, conferring resistance to sulphonamides, streptomycin and ampicillin by the presence of the *sul2-aph3-aph6-bla*_TEM-1_ genes, respectively [51].

Our findings highlight the potential risk of *mcr-1.1* + *mcr-5.1* spread among *Salmonella* spp. through IncHI2 plasmid, the large plasmid that carries multiple antibiotic and heavy metal resistance genes and may confer to strains a great survival advantage under unfavourable conditions [48,52]. Co-existence of *mcr-1* and *mcr-3* genes, on a 61 kb IncP plasmid, on a large plasmid of unknown incompatibility group or in a hybrid plasmid containing IncHI1-IncN replicons was identified in *E. coli* from cattle, pigs and poultry, in Spain and China, respectively [53,54]. The co-occurrence of *mcr-1 + mcr-5* and *mcr-4 + mcr-5* genes was reported in enteropathogenic *E. coli* from swine farms in Spain. However, a plasmid study was not performed [55]. Up to date, the co-presence of these two *mcr-1.1 + mcr-5.1* determinants in the same plasmid has not been previously described.

The identification of the *mcr-1.1* gene in a *S*. Enteritidis isolated in 2009 in humans is of main concern. This result traces back to 2009, the earliest report of *mcr* genes in *Salmonella* in Italy. The p61/4/09 *mcr-1.1*-IncX4 plasmid found in *S*. Enteritidis was closely related to the pMCR1.2-IT *mcr-1*-IncX4 plasmid (KX236309). The *mcr-1* and *pap2* genes lacked both copies of IS*Apl1* and the putative inverted repeat sequences, suggesting that it is not mobilisable in this configuration. *mcr-1* has been frequently associated with IncX4 plasmids in Enterobacterales isolated from humans, animals and products of animal origin in many countries like China, the United Kingdom, Spain, Portugal or Italy [31,56,57,58,59,60]. The IncX4 plasmids play an important role in spreading the *mcr-1* gene.

The absence of MICs differences observed in the two *mcr* positive COL-R strains with one or two *mcr* genes could be explained by several factors, including the low or high copy number of the plasmid carrying the *mcr* determinant, the genetic background of the host strain, the *Salmonella* serotype or the *mcr* promoter functionality [25,61,62].

It is worth noting the high percentage of COL resistance and the low presence of *mcr* observed in the *S*. Enteritidis serotype, one of the prevalent human serotypes in Europe and the main serotype responsible for infections in chickens [39]. This serotype belongs to the serogroup D and is characterised worldwide for predisposition to resistance to COL [63,64]. Some COL resistance mechanisms have been described [17,65,66,67], but others still have to be characterised [68,69]. In our study, six mutated proteins were selected as candidates for conferring the COL-R phenotype in the *mcr*-negative COL-R *S*. Enteritidis strains. The genes coding these proteins were identified in clusters that were previously described to be implicated in lipid A modification or synthesis or within pathways that were linked with COL MIC increment in *S. enterica* or other GNB species:

1. The rhamnosyltransferase RfbN protein is one of the key factors of the O-antigen biosynthesis and showed the deleterious D107V mutation in the COL-R *S.* Enteritidis 45/7/18 (Table 2). The O-antigen oligosaccharide of *S*. Enteritidis and *S*. Typhimurium contains rhamnose [70]. The rhamnosyltransferases are identical in *Salmonella* groups A, B, D1 and D2 [71,72]. Proteins involved in the biosynthesis of the basic O-antigen, present in the same operon of RfbN, have been previously involved in conferring COL resistance [17].
2. The R26L deleterious mutation found in the 45/7/18 strain involves the transcriptional regulatory protein ZraR activated by the sensor kinase ZraS in a zinc-dependent response regulation of a TCS (Table 2). It has been related to envelope environmental stress response, metabolism, protein synthesis, motility and biofilm formation. ZraR is also a bacterial enhancer-binding protein, controlling the multidrug export proteins AcrE, MdtE and MdtL. ZraR controls also LPS synthesis by RfaD and CpxP, the chaperone and modulator of CpxAR, respectively, that are directly involved in COL resistance [6,73]. CpxP-superfamily plays a role in resistance against polymyxin B in *S. enterica* [74]. Mutations in genes encoding regulators are essential in the adaptation process since mutations in regulatory elements can affect a broad range of targets [75].
3. *S*. Enteritidis 58/10/16 showed a deleterious mutation R298C in MdsC, an outer membrane lipoprotein, part of the MdsAB-MdsC tripartite efflux pump, one of the efflux pump systems of the RND family (Table 2). The mutation found in this efflux pump component may result in overproduction of the pump or have other effects leading to COL resistance. In *P. aeruginosa*, the overexpression of the homolog RND efflux pump MexAB-OprM has been linked to COL resistance [76]. However, this mutation was not causing a significant increase in resistance to other antimicrobial compounds than COL.
4. The deleterious mutation S91R, in *S*. Enteritidis 58/10/16 strain was detected in the outer membrane lipoprotein receptor LolB (Table 2). It is part of the system that transports lipoproteins, directly involved in outer membrane biogenesis and essential for cell viability [77]. A COL-R LPS-deficient *Acinetobacter baumannii* strain increased the exopolysaccharide production and the expression of *lol* genes to compensate for its deficit [78].
5. The deleterious mutation R127L in the YdeI protein was observed in the 58/10/16 strain (Table 2). It encodes an oligosaccharide/oligonucleotide binding-fold (OB-fold) protein important for polymyxin B resistance. It is regulated by the RcsBCD, PhoP-PhoQ and PmrA-PmrB sensor-kinase systems that modify gene expression in the presence of cationic antimicrobial peptides (CAMPs) in *S. enterica*. Furthermore, it could interact with OmpD in the outer membrane to facilitate CAMP resistance [79].
6. In the 58/10/16 strain, the Type IV pilus biogenesis protein PilN was also mutated (Table 2). It is part of the PilMNOP operon, promoting surface-associated twitching motility and virulence. In *P. aeruginosa,* it has been hypothesised that any mutation in PilN can destabilise the PilM-PilN interaction causing functionally significant structural changes in PilM [80,81]. In polymyxin B/COL-heteroresistant subpopulations colonies of *Neisseria meningitidis*, point mutations in *pilM* or *pilQ* were associated with the resistant phenotype [80]. Even if a direct relationship between these mutations and the COL resistance phenotype must be investigated, an association of different mutations in the same organism promoted a COL-R pattern. In *P. aeruginosa*, it has been asserted that the evolution of resistance is a complex, multistep process that requires a mutation in at least five independent loci that synergistically create the COL-R phenotype [75].

The evidence that four of these six mutated genes were within the cgMLST scheme strengthens the importance of the selected candidates. These mutated alleles are underrepresented in the proteins encoded within the pubMLST isolates collection and in the NCBI protein database. It suggests that these mutations are probably associated with COL-mediated positive selection and not for the result of a random, neutral genetic drift.

Functional studies, by site-direct mutagenesis or complementation assays, are needed to validate the role of these mutations in conferring resistance to COL.

Since mutations in genes encoding hypothetical proteins and mutations occurring in intergenic regions, including those caused by insertion sequence-integration, were not investigated in this study, we cannot exclude other unidentified mechanisms that may contribute to the COL resistance phenotype observed in these strains.

Both plasmid and chromosomal mutation conferring resistance impair bacterial fitness or cause fitness cost such as reducing growth, virulence or transmission [82]. Several studies revealed that COL resistance in *A. baumannii,* due to mutations in *pmrAB,* showed no loss of fitness and virulence; instead, COL resistance due to mutations in *lpx* genes, causing impaired LPS, suffered from a fitness cost and reduced virulence [83,84]. It has been demonstrated that COL-R *E. coli,* due to chromosomal mutations, has a fitness cost based on in vitro competition assays, although they did not show defects in growth rate. Mutations and altered expression in *phoPQ* or *pmrAB* and *eptA,* facilitating the addition of PEtN to lipid A of LPS, are a fitness burden on *E. coli,* explaining the low fitness of COL-R strains due to chromosomal mutations and the slow increase of COL resistance in GNB bacteria [85].

Antibiotic resistance mediated by acquiring mobile elements, including plasmids, imposes fitness costs on the bacterial host. In the absence of antibiotic pressure, sensitive strains can easily replace the resistant strains having an extra burden [86,87]. A recent study revealed that the expression of MCR-1 and MCR-3 has fitness cost on the first 50 generations of bacteria and that these genes and plasmids can persist over time, suggesting that the burden costs are alleviated through compensatory mutations [88]. Moreover, a study of the impact of diverse plasmids, harbouring COL resistance gene *mcr-1* on host fitness, demonstrated that the IncI2, IncHI2 and IncX4 plasmid-carrying *mcr-1* genes are stable and hardly affect bacterial growth, suggesting that most reported *mcr-1*-plasmids belong primarily to these types [88,89]. Increased fitness or co-selection by other antimicrobials might contribute to the further dissemination of *mcr-1*–positive plasmids [87].

Chromosomally mediated COL resistance is predominantly described in human clinical Enterobacterales isolates. Their prevalence will increase, especially in human medicine, where COL is increasingly used as a last-line antimicrobial treatment for carbapenemases-producing pathogens [2].

With the high use of COL in animal medicine, the increase of chromosomal-mediated COL resistance and the diffusion of mobile COL resistance mechanisms could also be expected, and the zoonotic agents *S*. Enteritidis and *S*. Typhimurium monophasic variant, respectively, are a clear example. A One Health approach for routine monitoring the COL resistance in humans, animals, food and environment and a moderated and controlled use of this class of antimicrobials in animal and human medicine will be one of the most important ways to contain the diffusion of this resistance. Several studies have been performed on drug repurposing in human medicine for carbapenem and COL-R GNB describing repurposed compounds (antiretrovirals, anticancer, antidepressants, antipsychotics, antiparasitic drugs or natural compounds). Furthermore, drug combination therapies (association of two or more drugs) can successfully increase the therapy’s efficacy. Various strategies have also been considered as treatment using faecal microbiota, antimicrobial peptides or bacteriophages. Additionally, studies on the CRISPR/Case9 system could also contribute to fighting antimicrobial resistance [90]. Moreover, alternatives to the use of COL in veterinary have been proposed. Depending on the resistance situation in a particular country, antimicrobial alternatives are aminopenicillins, trimethoprim-sulphonamides, tetracyclines, aminoglycosides, cephalosporins and fluoroquinolones. However, increasing resistance to those antibiotics is also of special concern. To avoid the use of antimicrobials and to promote global food security and global health security in food-producing animals, also many types of research have been focused on finding other alternatives such as vaccination, bacteriophage-based products, newly engineered enzyme-based experimental therapeutics, faecal transplant, small interfering RNAs, therapeutic antibodies, immune enhancers, probiotics, prebiotics, peptides, phytochemicals and heavy metals [91,92] (https://www.ars.usda.gov/alternativestoantibiotics/, accessed on 9 December 2021). Other interventions in the veterinary field should be encouraged to reduce the use of antimicrobials in animals, such as good farming practices and herd management, particularly by cleaning and disinfection strategies (biocides) or promoting an animal quarantine or restrictions on movements before freedom of disease certification [92].

## 4. Materials and Methods

### 4.1. Settings and Bacterial Isolates

Surveillance of *Salmonella* spp. isolates in humans in Italy is based on a dedicated network, Enter-Net Italia, coordinated by the Infectious Disease Department of the Istituto Superiore di Sanità. A retrospective study, which included a 3-year period (2016–2018), was performed to assess the prevalence of COL resistance in human *S. enterica* isolates. About 313 (20.5%) human-epidemiologically unrelated *S*. *enterica* strains were randomly selected from the Enter-Net Italia surveillance 2016–2018 collection. They belonged to 43 different serotypes: 104 *S*. Enteritidis, 64 *S*. Typhimurium monophasic variant (*S*. 1,4,[5],12:i:-), 26 *S*. Napoli, 23 *S*. Brandenburg, 18 *S*. Typhimurium, 11 *S*. Infantis, 7 *S*. Typhi, 7 *S*. Rissen and 53 of other serogroups (Supplementary Table S1). *S. enterica* strains were mainly isolated from faeces (282), 16 from blood, nine from urine and six from stools of unknown provenience (Supplementary Table S1). One *mcr-1* positive *S*. Enteritidis strain (61/4/09) isolated by the Enter-Net Italia surveillance in 2009, previously to the monitored period (data not shown), was added to the study for WGS analysis (Table 1). Since no confidential patient information was used and an ethics statement was not applicable, Institutional Review Board approval or patients’ informed consent was not required.

### 4.2. Antimicrobial Susceptibility Testing and Detection of Mcr Genes

Susceptibility to COL was determined in all the *S. enterica* strains by the reference broth microdilution method, as recommended by the European Committee on Antimicrobial Susceptibility Testing (EUCAST) [93]. COL (sulphate salt; Sigma-Aldrich, St. Quentin Fallavier Cedex, France) was used in a serial two-fold dilution ranging from 0.25 mg/L to 16 mg/L. About 212/313 (67.7%) randomly selected strains were also tested for a 16 antimicrobials susceptibility panel using the disk diffusion method with antimicrobial discs (Becton Dickinson, Sparks Glencoe, MD, USA). The antibiotic amount (μg) was as follows: nalidixic acid (NA, 30), pefloxacin (PEF, 5) ampicillin (A, 10), cefotaxime (CTX, 5), ceftazidime (CAZ, 10), cefoxitin (FOX, 30), amoxicillin/clavulanic acid 2:1 (AMC, 20/10), meropenem (MEM, 10), chloramphenicol (C, 30), gentamicin (G, 10), kanamycin (K, 30), streptomycin (S, 10), sulphonamides (Su, 0.25), tetracycline (T, 30), trimethoprim (TMP, 5) and trimethoprim-sulfamethoxazole (SXT, 1.25/23.75). The control strains were ATCC 25922 and NCTC 13864 *mcr-1*-positive *E. coli* strains. The susceptibility data were interpreted using the EUCAST guidelines (https://www.eucast.org/fileadmin/src/media/PDFs/EUCAST_files/Breakpoint_tables/v_9.0_Breakpoint_Tables.pdf, accessed on 15 September 2019).

All the COL-R strains were subjected to PCR to detect plasmid-mediated transferable *mcr-1* to *mcr-10* resistance determinants [94,95].

### 4.3. Whole-Genome Sequencing (WGS)

Genomic DNAs were purified using the NucleoSpin Tissue kit (Macherey-Nagel, Duren, Germany). DNA libraries were created using the Nextera XT DNA Library preparation kit (Illumina, San Diego, CA, USA). Sequencing was performed on the MiSeq platform according to the 2 × 300 PE protocol (Illumina, San Diego, CA, USA). *De novo* assembly of Illumina reads was performed using the SPAdes (Galaxy Version 3.11.1 software) and in parallel with the A5 pipeline (Galaxy Version 20150522) at the https://w3.iss.it/site/aries/, accessed on 26 July 2019). Plasmids sequences were manually curated, and PCR was performed to close and confirm the plasmid backbone (data not shown). Genome sequences were annotated at the RAST server (http://rast.nmpdr.org/, accessed on 10 October 2020). Annotation of plasmid sequences was obtained by Prokka Prokaryotic genome annotation (Galaxy Version 1.13) and manually curated by Sequin Application version 16.0 annotation software. Plasmid and resistance gene content was obtained using PlasmidFinder and ResFinder tools (https://cge.cbs.dtu.dk/services/, accessed on 21 July 2021), respectively. AMRFinderPlus, developed by NCBI [96], was also used to identify AMR genes, resistance-associated point mutations and metal resistance genes. Replicon alleles were assigned at the plasmid Multi Locus Sequence Typing (pMLST) site (https://pubmlst.org/plasmid/, accessed on 21 July 2021). Serotype was confirmed by SerotypeFinder 2.0 (https://cge.cbs.dtu.dk/services/, accessed on 21 July 2021). Core Genome MLST (cgMLST) was performed at the site cgMLSTFinder 1.1 site (https://cge.cbs.dtu.dk/services/cgMLSTFinder, accessed on 1 November 2021) using the Enterobase scheme [97]. Phylogenomic analysis was established through the CSI Phylogeny pipeline, calling and filtering SNPs, doing site validation and inferring a phylogeny based on the concatenated alignment of the high-quality SNPs [98]. SNPs phylogenetic tree was constructed and visualised by the FigTree v1.4.4 software.

Genomic analysis of COL-R *mcr*-negative *S*. Enteritidis strains was performed, using BLAST 2 Sequences for nucleotide sequence comparison (https://blast.ncbi.nlm.nih.gov/Blast.cgi, accessed on 25 July 2021), on previously described key genes related to COL resistance in GNB [17,18,67,99]. An additional genomic investigation was performed analysing genome synteny between COL-R *mcr*-negative *S*. Enteritidis strains against the COL-S *S*. Enteritidis strains by SEED viewer version 2.0 [100] (http://rast.nmpdr.org/seedviewer.cgi, accessed on 25 May 2020).

The functional impact of single-nucleotide-variants (SNVs) identified in mutated protein sequences was predicted using PROVEAN (Protein Variation Effect Analyzer) online software tool (http://provean.jcvi.org/seq_submit.php, accessed on 10 August 2021) [101]. Variants with a score equal to or below −2.5 were considered ‘deleterious’, and variants with a score above −2.5 were considered ‘neutral’ [101]. Neutral mutated proteins were not further considered in this study. In COL-R *mcr*-negative *S*. Enteritidis strains, the genes encoding deleterious mutated proteins previously described as correlated for COL MIC resistance in *S*. *enterica* or other GNB species were selected and compared with those localised within the cgMLST pubMLST site [29]. Furthermore, the deleterious mutated proteins identified in COL-R *mcr*-negative *S*. Enteritidis strains were compared with *S*. Enteritidis WGSs available at the NCBI GenBank by BLASTP (https://blast.ncbi.nlm.nih.gov/Blast.cgi, accessed on 1 November 2021).

## Figures and Tables

**Table 1:** Genetic and phenotypic characteristics of the four COL-R *S. enterica* isolates analysed in this study compared with COL-S strains isolated in the same 2016–2018 period and with the COL-R *mcr-1* positive strain isolated in 2009.

Strain	Serogroup	Isolation Year	MIC (mg/L)	MLST (ST)	Resfinder Detected Acquired Antimicrobial-Resistant Genes	Mutations in the Chromosome of COL-R Strains ^a^	ResFinder Detected Mutations in COL-R Strains ^b^	Metal Resistant Genes	Plasmid Finder [pMLST/pDLST/ FAB] ^c^	Genbank acc. Number (*mcr* Plasmid acc. Number) ^c^
58/10/16	*S*. Enteritidis	2016	4	ST3645	neg	MdsC R298C PilN P107L YdeI R127L LolB S91R	neg	*golTS*	IncFII(S), IncFIB(S) [S1:A-:B22]	SAMN13046498
83/46/17	*S*. Enteritidis	2017	4	ST3233	neg	wt	neg	*golTS*	IncFII(S), IncFIB(S) [S1:A-:B22]	SAMN13046552
45/7/18	*S*. Enteritidis	2018	4	ST11	neg	ZraR R26L RfbN D107V	neg	*golTS*	IncFII(S) IncFIB(S) [S1:A-:B22]	SAMN13047690
77/84/18	*S*. Typhimurium monophasic var.	2016	4	ST34	*mcr-1.1, mcr-5.1, sul2, aph(6)-Id, aph(4)-Ia, aac(3)-I v, aph(3’’)-Ib,* *tet(M)-like, bla*_TEM-1_	nd	neg	*golTS, pcoSRDCA, silPABFCRSE, arsCBADRST, terWZD*	IncHI2, IncHI2A, IncQ1 [ST-4] ColRNAI	SAMN13046551 (MZ666126)
56/1/16	*S*. Enteritidis	2016	2	ST11	neg	wt	neg	*golTS*	IncFII(S), IncFIB(S) [S1:A-:B22]	SAMN13046483
4/23/16	*S*. Enteritidis	2016	1	ST11	neg	wt	neg	*golTS*	IncFII(S), IncFIB(S) [S1:A-:B22]	SAMN13039343
61/4/09	*S*. Enteritidis	2009	4	ST11	*mcr-1.1, sul2, dfrA14, aph(3′′)-Ib,aph(6)-Id,* *tet(A), bla*_TEM-1_	nd	GyrA_D87Y	*golTS*	IncFII(S), IncFIB(S) [S1:A-:B22] IncX4 IncN [ST-3]	SAMN13046518 (OK605084)

^a^ Mutated proteins identified in the genomes of three COL-R *S.* Enteritidis strains (58/10/16; 83/46/17; 45/7/18) in comparison with the genomes of the two COL-S strains from the same collection (56/1/16; 4/23/16); ^b^ Chromosomal point mutations conferring resistance determined by ResFinder tool (https://cge.cbs.dtu.dk/services/, accessed on 25 May 2021). wt: wild type, no mutated proteins detected; nd: not determined, chromosomal mutations were not investigated in the *mcr*-positive strains; neg: negative. ^c^ Underlined replicons and accession numbers refer to the location of *mcr* genes on the respective plasmids.

**Table 2:** WGS characterisation of deleterious mutated proteins potentially related with COL resistance in COL-R *mcr* negative *S*. Enteritidis strains.

Protein Name	PROVEAN	58/10/16	83/46/17	45/7/18
Multidrug efflux system, outer membrane factor lipoprotein of OprM/OprM family, MdsC	−7.963	R298C	no mutation	no mutation
Type IV pilus biogenesis protein, PilN	−9.667	P107L	no mutation	no mutation
Yde family stress tolerance OB-fold protein, YdeI	−2.914	R127L	no mutation	no mutation
Outer membrane lipoprotein component of the lipoprotein transport system, LolB	−3.279	S91R	no mutation	no mutation
Response regulator of zinc sigma-54-dependent two-component system, ZraR	−2.709	no mutation	no mutation	R26L
O antigen biosynthesis rhamnosyltransferase, RfbN	−7.898	no mutation	no mutation	D107V

No mutation: No mutation was detected in the protein sequence compared with the COL-S *S*. Enteritidis 4/23/16 and 56/1/16 strains.

## Data Availability

Genomic and plasmidic sequences have been deposited in GenBank (https://www.ncbi.nlm.nih.gov/pubmed, accessed on 17 October 2019) under BioProject accession number PRJNA578046. Strains have been stored under the BioSample accession numbers: SAMN13039343, SAMN13046483, SAMN13046498, SAMN13046552, SAMN13047690, SAMN13046518, SAMN13046551 (Table 1). Manually curated plasmid sequences were released under accession numbers: OK605084 and MZ666126 (Table 1).

## Supplementary Materials

The following are available online at https://www.mdpi.com/article/10.3390/antibiotics11010102/s1, Supplementary Figure S1: Single nucleotide polymorphism and core genome MLST analysis performed COL-S and COL-R *mcr*-negative *Salmonella enterica* serovar Enteritidis genomes analysed in this study. Supplementary Table S1: Epidemiological data and antimicrobial resistance of all the strains included in this study. Supplementary Table S2: Deleterious mutated proteins present in COL-R *mcr*-negative *S*. Enteritidis strains were not further discussed in the study. Supplementary Table S3: Analysis of *pilN*, *ydeI*, *lolB* and *zraR* allele frequency in COL-R *mcr*-negative *S.* Enteritidis strains against wild type alleles in the cgMLST database (pubMLST site).
