# Supplementary material for: Colistin Resistance Mechanisms in Human Salmonella enterica Strains Isolated by the National Surveillance Enter-Net Italia (2016–2018)
Source: Antibiotics (Basel). 2022 Jan 13;11(1):102. doi: 10.3390/antibiotics11010102 (PMC8772777; doi:10.3390/antibiotics11010102)
Supplement: Supplementary file 1 [file antibiotics-11-00102-s001.zip › Supplementary Figure S1.pdf]

Supplementary Figure S1. **Single Nucleotide Polymorphism and core genome MLST analysis performed COL-S and COL-R *mcr*-negative *Salmonella enterica* serovar Enteritidis genomes analyzed in this study.**

Panel A: SNP-based phylogenetic tree created by CSIPhylogeny DTU tool.

Panel B: SNP Matrix obtained by CSIPhylogeny DTU tool.

Panel C: cgMLST matrix created by DTU tool.

(A)

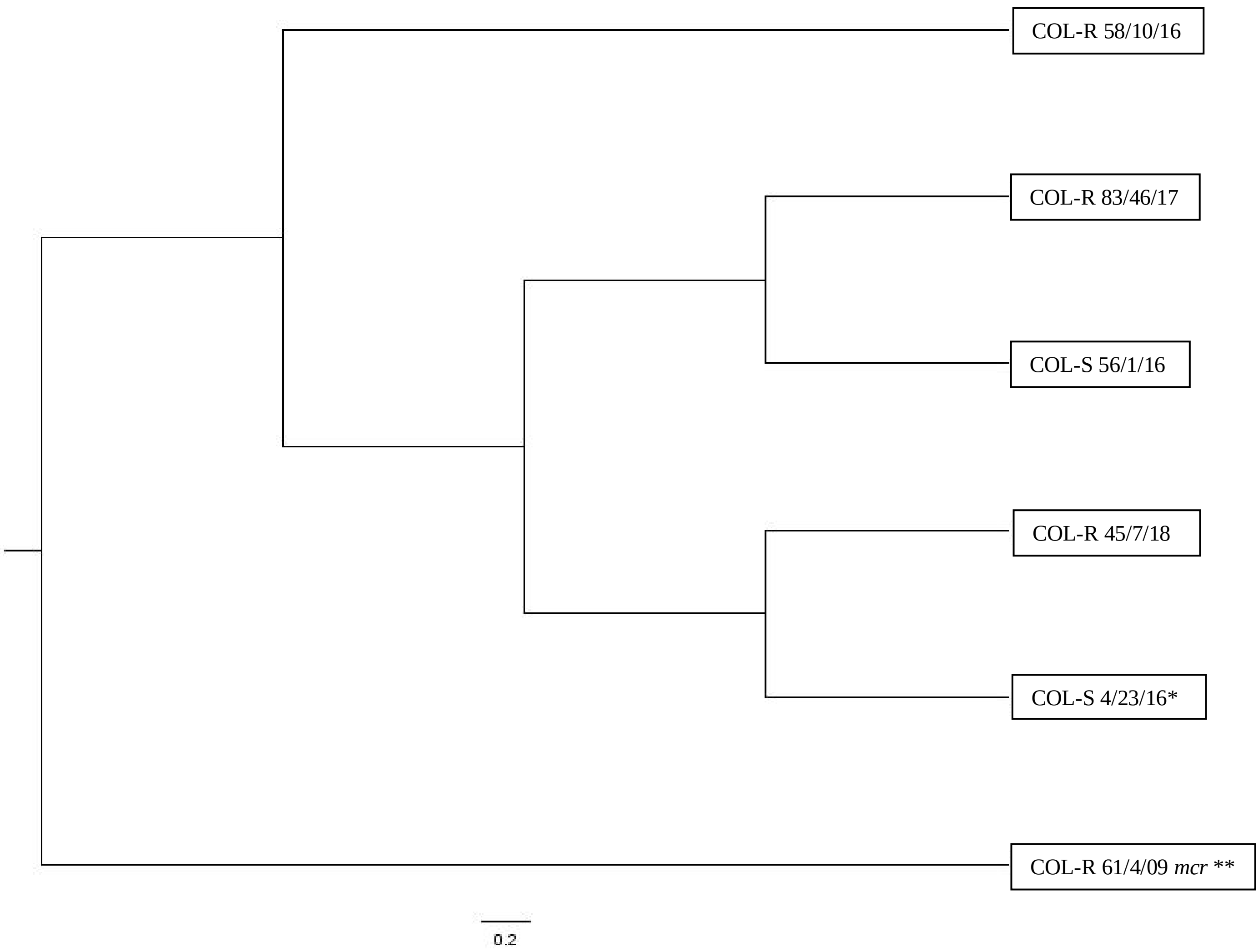

(B)

SNP matrix[a]	COL-R *mcr* 61/4/09**	COL-R 45/7/18	COL-R 58/10/16	COL-R 83/46/17	COL-S 4/23/16*	COL-S 56/1/16
61/4/09	0	494	494	479	487	491
45/7/18	494	0	97	44	59	58
58/10/16	494	97	0	79	94	91
83/46/17	479	44	79	0	45	36
4/23/16	487	59	94	45	0	57
56/1/16	491	58	91	36	57	0

(C)

cgMLST matrix[b]	COL-R *mcr* 61/4/09*	COL-R 45/7/18	COL-R 58/10/16	COL-R 83/46/17	COL-S 4/23/16	COL-S 56/1/16
61/4/09	0	221	222	214	219	218
45/7/18	221	0	43	24	31	30
58/10/16	222	43	0	35	44	40
83/46/17	214	24	35	0	25	17
4/23/16	219	31	44	25	0	28
56/1/16	218	30	40	17	28	0

In Panel A, * COL-S *S.* Enteritidis 4/23/16 was chosen as reference strain for the SNP analysis. ** Unrelated COL-R *mcr*-positive *S.* Enteritidis was added for comparison.

In Panel B [a] : Percentage of reference genome covered by all isolates: 98.9%. 4,651,340 positions were found in all analyzed genomes. Size of reference genome: 4,702,777.

In Panel B [b]: *Salmonella* core genome = 3,002 loci. cgMLST allele calls were accepted for allele coverage >95%.
