# Supplementary material for: Colistin Resistance Mechanisms in Human Salmonella enterica Strains Isolated by the National Surveillance Enter-Net Italia (2016–2018)
Source: Antibiotics (Basel). 2022 Jan 13;11(1):102. doi: 10.3390/antibiotics11010102 (PMC8772777; doi:10.3390/antibiotics11010102)
Supplement: Supplementary file 1 [file antibiotics-11-00102-s001.zip › Supplementary Table S1.pdf]

Supplementary Table S1. Epidemiological data and antimicrobial resistance of all the strains included in this study.

Strain	Serogroup	Sample	Date	MIC COL (mg/L)	PCR *mcr* 1-10/ Sequenced	Other antimicrobial resistance
61/4/09*	Enteritidis	UNK	10/09/2009	4	*mcr-1.1*/SEQ	ASuTNaSxt
c	1,4,[5],12:i:-	FAECES	21/10/2016	4	*mcr-1.1*+*mcr-5.1*/SEQ	ASSuTG
22/17/16	1,4,[5],12:i:-	FAECES	19/02/2016	4	neg	ASSuTAmc
57/13/16	Typhimurium	FAECES	05/07/2016	4	neg	ASSuTmpSxtAmc
57/14/16	Enteritidis	FAECES	24/07/2016	4	neg	ND
46/32/16	Enteritidis	FAECES	15/08/2016	4	neg	ND
53/13/16	Enteritidis	FAECES	20/09/2016	4	neg	ND
54/4/16	Enteritidis	FAECES	26/09/2016	4	neg	ND
54/11/16	Enteritidis	FAECES	07/10/2016	4	neg	ND
58/10/16	Enteritidis	FAECES	17/10/2016	4	neg/SEQ	Suscept
18/12/17	Enteritidis	FAECES	22/02/2017	4	neg	ND
18/16/17	Enteritidis	FAECES	01/03/2017	4	neg	ND
83/13/17	Enteritidis	UNK	16/03/2017	4	neg	Suscept
23/22/17	Enteritidis	FAECES	23/03/2017	4	neg	ND
49/10/17	Enteritidis	FAECES	03/07/2017	4	neg	ND
83/37/17	Enteritidis	FAECES	21/08/2017	4	neg	Suscept
57/8/17	Enteritidis	FAECES	22/08/2017	4	neg	ND
60/6/17	Enteritidis	FAECES	28/08/2017	4	neg	ND
83/47/17	Enteritidis	FAECES	15/09/2017	4	neg	Suscept
83/46/17	Enteritidis	FAECES	19/09/2017	4	neg/SEQ	Suscept
78/17/17	Enteritidis	FAECES	06/11/2017	4	neg	ND
17/1/18	Enteritidis	FAECES	02/02/2018	4	neg	Suscept
31/1/18	Enteritidis	FAECES	03/04/2018	4	neg	NaPef
75/20/18	Enteritidis	FAECES	02/05/2018	4	neg	Suscept
45/7/18	Enteritidis	FAECES	31/05/2018	4	neg/SEQ	Suscept
27/1/16	Enteritidis	FAECES	25/01/2016	2	neg	Suscept
27/10/16	Enteritidis	FAECES	11/03/2016	2	neg	Suscept
56/1/16	Enteritidis	FAECES	02/04/2016	2	neg/SEQ	Suscept
57/21/16	Enteritidis	FAECES	24/07/2016	2	neg	Suscept
57/28/16	Enteritidis	FAECES	14/08/2016	2	neg	Suscept
57/32/16	Enteritidis	FAECES	19/08/2016	2	neg	NaPef
57/39/16	Enteritidis	FAECES	06/09/2016	2	neg	Suscept
77/83/18	Enteritidis	FAECES	13/09/2016	2	neg	ND
57/41/16	Enteritidis	FAECES	18/09/2016	2	neg	Suscept
57/45/16	Enteritidis	FAECES	06/10/2016	2	neg	Suscept
55/20/16	Saintpaul	FAECES	09/08/2016	2	neg	ND
9/5/17	Enteritidis	FAECES	09/01/2017	2	neg	ND
18/11/17	Enteritidis	FAECES	21/02/2017	2	neg	ND
18/15/17	Enteritidis	FAECES	27/02/2017	2	neg	ND
23/17/17	Enteritidis	FAECES	11/03/2017	2	neg	ND
23/19/17	Enteritidis	FAECES	16/03/2017	2	neg	ND
23/18/17	Enteritidis	BLOOD	18/03/2017	2	neg	ND
23/25/17	Enteritidis	FAECES	20/03/2017	2	neg	ND

23/44/17	Enteritidis	FAECES	27/03/2017	2	neg	ND
53/1/17	Enteritidis	FAECES	21/07/2017	2	neg	ND
57/7/17	Enteritidis	FAECES	08/08/2017	2	neg	ND
83/32/17	Enteritidis	FAECES	09/08/2017	2	neg	Suscept
57/6/17	Enteritidis	BLOOD	16/08/2017	2	neg	ND
57/9/17	Enteritidis	FAECES	28/08/2017	2	neg	ND
60/13/17	Enteritidis	FAECES	02/09/2017	2	neg	ND
61/9/17	Enteritidis	FAECES	04/09/2017	2	neg	ND
60/15/17	Enteritidis	FAECES	06/09/2017	2	neg	ND
60/14/17	Enteritidis	FAECES	07/09/2017	2	neg	ND
53/32/17	1,4,[5],12:i:-	FAECES	26/07/2017	2	neg	ASSuT
53/36/17	1,4,[5],12:i:-	FAECES	27/07/2017	2	neg	ACSSuTTmpSxt
57/13/17	1,4,[5],12:i:-	FAECES	05/08/2017	2	neg	ASSuT
57/11/17	1,4,[5],12:i:-	FAECES	07/08/2017	2	neg	SSuT
53/35/17	1,4,[5],12:i:-	FAECES	17/08/2017	2	neg	ASSuT
60/11/17	1,4,[5],12:i:-	FAECES	06/09/2017	2	neg	ASSuT
78/8/17	1,4,[5],12:i:-	UNK	07/09/2017	2	neg	ASSuT
78/10/17	1,4,[5],12:i:-	UNK	15/09/2017	2	neg	ASSuT
78/9/17	1,4,[5],12:i:-	UNK	21/09/2017	2	neg	ASSuT
75/14/18	1,4,[5],12:i:-	FAECES	18/12/2017	2	neg	ASSuNaPef
13/1/18	Bovismorbificans	FAECES	12/10/2017	2	neg	Su
83/53/17	Brandenburg	FAECES	01/10/2017	2	neg	ND
51/73/18	Brandenburg	FAECES	22/10/2017	2	neg	Suscept
84/22/17	Brandenburg	FAECES	20/11/2017	2	neg	ND
83/22/17	Muenchen	BLOOD	07/06/2017	2	neg	Su
2/4/18	Thompson	FAECES	03/08/2017	2	neg	Suscept
8/15/18	Newport	FAECES	10/08/2017	2	neg	Suscept
45/28/18	Enteritidis	FAECES	21/01/2018	2	neg	ANaPef
52/2/18	Enteritidis	FAECES	08/03/2018	2	neg	ND
77/85/18	Enteritidis	FAECES	11/04/2018	2	neg	ND
52/5/18	Enteritidis	FAECES	26/04/2018	2	neg	ND
52/3/18	Enteritidis	BLOOD	28/04/2018	2	neg	ND
52/4/18	Enteritidis	FAECES	30/04/2018	2	neg	ND
52/6/18	Enteritidis	FAECES	04/05/2018	2	neg	ND
52/7/18	Enteritidis	FAECES	17/05/2018	2	neg	Suscept
52/8/18	Enteritidis	FAECES	24/05/2018	2	neg	Su
45/11/18	Enteritidis	FAECES	29/05/2018	2	neg	Suscept
45/8/18	Enteritidis	FAECES	31/05/2018	2	neg	Suscept
68/25/18	Enteritidis	FAECES	15/06/2018	2	neg	Su
77/24/18	Enteritidis	FAECES	10/07/2018	2	neg	Suscept
52/10/18	Enteritidis	BLOOD	13/07/2018	2	neg	Su
77/36/18	Enteritidis	FAECES	24/07/2018	2	neg	NaPef
77/41/18	Enteritidis	FAECES	30/08/2018	2	neg	Su
91/11/18	Enteritidis	FAECES	30/08/2018	2	neg	Suscept
13/11/17	Enteritidis	FAECES	08/09/2018	2	neg	ND
13/14/19	Enteritidis	FAECES	15/09/2018	2	neg	Su
91/32/18	Enteritidis	UNK	18/09/2018	2	neg	Suscept
15/10/19	Enteritidis	FAECES	08/12/2018	2	neg	Su
87/22/18	1,4,[5],12:i:-	UNK	17/01/2018	2	neg	ASSuT

13/8/19	1,4,[5],12:i:-	FAECES	01/02/2018	2	neg		ASSuTTmpSxtNaPef
13/9/19	1,4,[5],12:i:-	FAECES	02/08/2018	2	neg		ASSuTSxt
45/3/18	Napoli	FAECES	17/05/2018	2	neg		Suscept
11/4/19	Napoli	FAECES	05/11/2018	2	neg		Suscept
29/1/18	Typhi	FAECES	21/03/2018	2	neg		Suscept
11/5/19	Winston	FAECES	29/10/2018	2	neg		Suscept
4/23/16	Enteritidis	FAECES	06/01/2016	1	neg/SEQ		Suscept
57/19/16	Enteritidis	FAECES	24/07/2016	1	neg		ND
57/20/16	Enteritidis	FAECES	24/07/2016	1	neg		ND
55/39/16	Enteritidis	FAECES	28/09/2016	1	neg		ND
55/34/16	Enteritidis	FAECES	03/10/2016	1	neg		Suscept
57/37/16	1,4,[5],12:i:-	FAECES	01/09/2016	1	neg		ASSuT
57/38/16	1,4,[5],12:i:-	FAECES	18/09/2016	1	neg		ACSSuTGTmpSxt
17/2/18	Anatum	URINE	14/06/2016	1	neg		T
27/11/17	Bovismorbificans	FAECES	16/03/2016	1	neg		Suscept
56/1/18	Brandenburg	FAECES	31/03/2016	1	neg		Suscept
54/1/18	Brandenburg	FAECES	10/06/2016	1	neg		Suscept
39/8/16	Brandenburg	FAECES	27/06/2016	1	neg		ND
57/11/16	Gallinarum	FAECES	07/07/2016	1	neg		Suscept
38/20/17	Infantis	FAECES	23/01/2016	1	neg		ND
38/9/17	Derby	FAECES	05/12/2016	1	neg		ND
38/4/17	Saintpaul	FAECES	08/10/2016	1	neg		ND
55/38/16	Napoli	FAECES	15/09/2016	1	neg		ND
38/6/17	Virchow	FAECES	02/11/2016	1	neg		ND
8/12/18	Enteritidis	FAECES	13/06/2017	1	neg		Suscept
18/19/17	1,4,[5],12:i:-	FAECES	12/01/2017	1	neg		ND
8/9/18	1,4,[5],12:i:-	FAECES	10/03/2017	1	neg		ASSuTAmc
8/11/18	1,4,[5],12:i:-	FAECES	05/05/2017	1	neg		ASSuTAmc
53/33/17	1,4,[5],12:i:-	FAECES	31/07/2017	1	neg		ASSuT
53/34/17	1,4,[5],12:i:-	FAECES	07/08/2017	1	neg		ASSuT
63/12/17	1,4,[5],12:i:-	FAECES	08/08/2017	1	neg		ASSuTTmpSxtKCtx
75/7/18	1,4,[5],12:i:-	FAECES	12/08/2017	1	neg		AC
57/10/17	1,4,[5],12:i:-	FAECES	21/08/2017	1	neg		ACSSuTTmpSxt
60/3/17	1,4,[5],12:i:-	FAECES	04/09/2017	1	neg		ASSuT
60/10/17	1,4,[5],12:i:-	FAECES	06/09/2017	1	neg		ASuT
78/5/17	1,4,[5],12:i:-	FAECES	16/10/2017	1	neg		ASSuT
78/7/17	1,4,[5],12:i:-	FAECES	18/10/2017	1	neg		TmpSxt
8/17/18	Typhimurium	FAECES	26/09/2017	1	neg		Suscept
8/21/18	Typhimurium	FAECES	17/11/2017	1	neg		Suscept
54/8/18	Brandenburg	FAECES	27/08/2017	1	neg		Suscept
83/49/17	Brandenburg	FAECES	25/09/2017	1	neg		ND
51/75/18	Brandenburg	FAECES	28/10/2017	1	neg		ND
84/20/17	Brandenburg	FAECES	13/11/2017	1	neg		ND
51/74/18	Brandenburg	FAECES	22/11/2017	1	neg		Suscept
54/20/18	Brandenburg	FAECES	15/12/2017	1	neg		Suscept
5/9/18	Brandenburg	FAECES	16/12/2017	1	neg		Suscept
45/4/18	6,7:l,w:-	FAECES	31/12/2017	1	neg		Suscept
75/13/18	Corvallis	FAECES	28/10/2017	1	neg		Su
83/1/17	Infantis	FAECES	09/01/2017	1	neg		ATSuNaPefSxtTmpKCtx

83/15/17	Infantis	FAECES	27/03/2017	1	neg	Suscept
4/25/19	Infantis	FAECES	05/04/2017	1	neg	ND
60/9/17	Infantis	FAECES	30/08/2017	1	neg	ND
40/7/17	Napoli	URINE	22/05/2017	1	neg	ND
75/3/18	Napoli	BLOOD	10/07/2017	1	neg	Suscept
66/5/17	Napoli	FAECES	27/07/2017	1	neg	ND
53/42/17	Napoli	FAECES	07/08/2017	1	neg	ND
53/43/17	Napoli	FAECES	12/08/2017	1	neg	ND
75/9/18	Napoli	FAECES	18/08/2017	1	neg	Suscept
72/4/17	Napoli	FAECES	28/09/2017	1	neg	ND
83/56/17	Napoli	FAECES	12/10/2017	1	neg	ND
75/10/18	Ndolo	FAECES	01/09/2017	1	neg	Suscept
8/19/18	Paratyphi b	FAECES	05/10/2017	1	neg	Su
45/16/18	Enteritidis	FAECES	21/03/2018	1	neg	Suscept
68/14/18	Enteritidis	FAECES	03/04/2018	1	neg	Suscept
91/5/18	Enteritidis	FAECES	18/07/2018	1	neg	Suscept
77/77/18	Enteritidis	FAECES	28/08/2018	1	neg	Suscept
77/21/18	Enteritidis	FAECES	31/08/2018	1	neg	Suscept
68/26/18	1,4,[5],12:i:-	FAECES	05/07/2018	1	neg	ASSuTAmc
77/45/18	1,4,[5],12:i:-	FAECES	04/09/2018	1	neg	ASSuTAmc
77/38/18	1,4,[5],12:i:-	FAECES	10/09/2018	1	neg	ASSuTAmc
91/23/18	1,4,[5],12:i:-	FAECES	12/09/2018	1	neg	ASSuT
77/61/18	1,4,[5],12:i:-	FAECES	13/09/2018	1	neg	ASSuT
3/9/19	1,4,[5],12:i:-	FAECES	30/10/2018	1	neg	ACSSuTTmpSxtKGNaPef
15/1/19	1,4,[5],12:i:-	FAECES	06/11/2018	1	neg	ASSuT
36/1/18	6,7:-:1,5	FAECES	22/03/2018	1	neg	ND
45/5/18	6,7:-:1,5	BLOOD	31/03/2018	1	neg	ACSTG
54/29/18	Brandenburg	FAECES	25/01/2018	1	neg	Suscept
51/76/18	Brandenburg	FAECES	15/02/2018	1	neg	Suscept
51/69/18	Brandenburg	FAECES	20/02/2018	1	neg	Suscept
51/67/18	Brandenburg	FAECES	16/04/2018	1	neg	Suscept
51/68/18	Brandenburg	FAECES	19/05/2018	1	neg	Suscept
51/71/18	Brandenburg	FAECES	24/05/2018	1	neg	Suscept
55/20/18	Infantis	FAECES	23/01/2018	1	neg	Suscept
45/19/18	Infantis	FAECES	15/03/2018	1	neg	ASSuTAmcSxtTmpKNaPef
4/28/19	Infantis	FAECES	31/05/2018	1	neg	ASSuTTmpSxtKCtxNaPef
73/2/18	Napoli	FAECES	11/06/2018	1	neg	Suscept
93/2/18	Napoli	FAECES	24/08/2018	1	neg	Suscept
18/3/19	Napoli	FAECES	06/09/2018	1	neg	Suscept
93/8/18	Napoli	FAECES	20/09/2018	1	neg	Suscept
4/29/19	Rissen	URINE	24/10/2018	1	neg	ATAmcKCazCtxFox
13/24/19	Typhi	BLOOD	02/10/2018	1	neg	ND
55/19/16	Typhimurium	FAECES	07/09/2016	1	neg	ND
57/43/16	Typhimurium	URINE	27/09/2016	1	neg	ACSSuTAmcNaPef
68/30/18	Typhimurium	FAECES	20/03/2018	1	neg	ACSSuTAmc
68/23/18	Typhimurium	FAECES	03/06/2018	1	neg	AAmc
3/1/19	Typhimurium	FAECES	21/07/2018	1	neg	ACSSuTPefCazCtx
45/2/18	Umbilo	FAECES	04/05/2018	1	neg	Suscept
58/37/18	Veneziana	FAECES	02/07/2018	1	neg	ND

75/16/18	Enteritidis	FAECES	08/01/2016	0.5	neg	Suscept
57/2/16	Enteritidis	FAECES	13/05/2016	0.5	neg	Suscept
57/31/16	Enteritidis	FAECES	09/07/2016	0.5	neg	Suscept
57/24/16	Enteritidis	FAECES	02/08/2016	0.5	neg	Suscept
57/40/16	Enteritidis	FAECES	14/09/2016	0.5	neg	Suscept
55/35/16	Enteritidis	FAECES	14/09/2016	0.5	neg	ND
55/41/16	Enteritidis	FAECES	16/09/2016	0.5	neg	ND
55/13/16	Enteritidis	FAECES	20/09/2016	0.5	neg	ND
57/42/16	1,4,[5],12:i:-	FAECES	19/09/2016	0.5	neg	ASSuT
57/9/16	Agama	FAECES	27/06/2016	0.5	neg	ASSuAmc
27/26/16	Bovismorbificans	FAECES	11/04/2016	0.5	neg	Su
55/12/16	Derby	FAECES	24/08/2016	0.5	neg	SSu
55/27/16	Napoli	FAECES	04/08/2016	0.5	neg	ND
27/19/16	Panama	FAECES	19/02/2016	0.5	neg	SuT
55/37/16	Panama	FAECES	30/09/2016	0.5	neg	ASuTAmcTmpSxt
27/8/16	Paratyphi a	BLOOD	04/03/2016	0.5	neg	NaPef
27/4/16	Rissen	FAECES	24/01/2016	0.5	neg	ASuTAmcTmpSxt
77/82/18	Schwarzengrund	FAECES	03/08/2016	0.5	neg	ND
55/18/16	Typhimurium	FAECES	02/08/2016	0.5	neg	ND
55/32/16	Typhimurium	FAECES	02/09/2016	0.5	neg	ND
55/11/16	Typhimurium	FAECES	10/09/2016	0.5	neg	ND
5/7/17	Enteritidis	FAECES	02/01/2017	0.5	neg	ND
8/13/18	Enteritidis	FAECES	13/06/2017	0.5	neg	Suscept
8/16/18	1,4,[5],12:i:-	FAECES	07/08/2017	0.5	neg	ASSuTAmc
60/1/17	1,4,[5],12:i:-	FAECES	07/08/2017	0.5	neg	ND
61/8/17	1,4,[5],12:i:-	FAECES	12/09/2017	0.5	neg	ASSu
78/4/17	1,4,[5],12:i:-	FAECES	15/10/2017	0.5	neg	TmpSxt
75/12/18	1,4,[5],12:i:-	FAECES	18/10/2017	0.5	neg	ASuT
78/11/17	1,4,[5],12:i:-	FAECES	23/10/2017	0.5	neg	ND
17/5/18	43:z4,z23:-	FAECES	15/10/2017	0.5	neg	SSu
51/72/18	Brandenburg	FAECES	13/11/2017	0.5	neg	Suscept
75/11/18	Corvallis	FAECES	19/09/2017	0.5	neg	Su
75/15/18	Livingstone	FAECES	21/12/2017	0.5	neg	Suscept
75/6/18	Napoli	BLOOD	13/08/2017	0.5	neg	Suscept
75/8/18	Napoli	FAECES	14/08/2017	0.5	neg	Suscept
2/1/18	Napoli	FAECES	25/09/2017	0.5	neg	Suscept
36/1/17	Paratyphi a	BLOOD	07/03/2017	0.5	neg	SuNaPef
83/18/17	Typhi	BLOOD	14/04/2017	0.5	neg	NaPef
83/58/17	Typhi	BLOOD	15/10/2017	0.5	neg	ACSSuAmcTmpSxtNaPef
8/14/18	Worthington	FAECES	07/07/2017	0.5	neg	Su
13/10/19	1,4,[5],12:i:-	FAECES	04/01/2018	0.5	neg	ASSuTSxtG
55/19/18	1,4,[5],12:i:-	FAECES	19/01/2018	0.5	neg	ASSuTAmc
13/16/19	1,4,[5],12:i:-	FAECES	22/01/2018	0.5	neg	ASSuTTmpSxtKNaPef
55/27/18	1,4,[5],12:i:-	FAECES	28/01/2018	0.5	neg	ASSuTAmc
68/8/18	1,4,[5],12:i:-	FAECES	05/03/2018	0.5	neg	ASSuT
68/31/18	1,4,[5],12:i:-	FAECES	09/04/2018	0.5	neg	ASSuTAmc
75/21/18	1,4,[5],12:i:-	FAECES	28/05/2018	0.5	neg	ASSuAmc
3/3/19	1,4,[5],12:i:-	FAECES	14/08/2018	0.5	neg	ASSuTAmc
3/5/19	1,4,[5],12:i:-	FAECES	24/08/2018	0.5	neg	ACSu

77/62/18	1,4,[5],12:i:-	FAECES	31/08/2018	0.5	neg	ASSuT
77/30/18	1,4,[5],12:i:-	FAECES	07/09/2018	0.5	neg	ASSuTAmc
3/8/19	1,4,[5],12:i:-	FAECES	14/09/2018	0.5	neg	ASSuT
13/12/19	1,4,[5],12:i:-	FAECES	20/09/2018	0.5	neg	SuT
79/15/18	1,4,[5],12:i:-	FAECES	04/10/2018	0.5	neg	ASSuT
13/15/19	1,4,[5],12:i:-	FAECES	18/10/2018	0.5	neg	ASSuTTmpSxt
15/3/19	1,4,[5],12:i:-	FAECES	15/11/2018	0.5	neg	ASSuT
15/13/19	1,4,[5],12:i:-	FAECES	27/12/2018	0.5	neg	ASSuT
13/22/19	41:z:1,5	FAECES	31/08/2018	0.5	neg	NaPef
55/22/18	Brandenburg	FAECES	22/01/2018	0.5	neg	Suscept
56/4/18	Brandenburg	FAECES	27/06/2018	0.5	neg	ND
13/1/19	Brandenburg	FAECES	11/08/2018	0.5	neg	ND
17/3/18	Butantan	FAECES	30/01/2018	0.5	neg	ASSuTAmc
13/7/19	Choleraesuis	BLOOD	06/10/2018	0.5	neg	ND
74/1/18	Coeln	FAECES	02/08/2018	0.5	neg	ND
13/5/19	Coeln	FAECES	05/11/2018	0.5	neg	ND
75/17/18	Cullingworth	FAECES	30/03/2018	0.5	neg	Su
13/13/19	Derby	FAECES	13/10/2018	0.5	neg	ASSuTTmpSxtGNaPef
4/14/19	Goldcoast	FAECES	02/08/2018	0.5	neg	Suscept
13/3/19	Goldcoast	FAECES	16/11/2018	0.5	neg	ND
4/26/19	Infantis	FAECES	15/03/2018	0.5	neg	ASSuTTmpSxtKCazCtxNaPef
93/1/18	Napoli	FAECES	21/08/2018	0.5	neg	Suscept
93/5/18	Napoli	FAECES	05/09/2018	0.5	neg	Suscept
47/1/18	Pasing	URINE	31/05/2018	0.5	neg	ND
75/18/18	Rissen	URINE	05/04/2018	0.5	neg	Suscept
13/18/19	Rissen	FAECES	22/10/2018	0.5	neg	ND
15/14/19	Rissen	FAECES	23/12/2018	0.5	neg	Su
45/6/18	*S*.spp	URINE	30/03/2018	0.5	neg	SuT
36/2/18	Tambacounda	URINE	21/03/2018	0.5	neg	ND
13/25/19	Typhi	BLOOD	13/06/2018	0.5	neg	ND
45/27/18	Typhimurium	FAECES	03/01/2018	0.5	neg	SSuT
55/24/18	Typhimurium	FAECES	12/01/2018	0.5	neg	ND
52/9/18	Typhimurium	FAECES	08/06/2018	0.5	neg	ASSuTAmcTmpSxtCazCtx
68/27/18	Typhimurium	FAECES	17/07/2018	0.5	neg	ASSuAmc
3/7/19	Typhimurium	FAECES	13/09/2018	0.5	neg	ND
13/20/19	Typhimurium	FAECES	08/11/2018	0.5	neg	ACSSuTAmcNaPef
45/25/18	Veneziana	FAECES	17/04/2018	0.5	neg	ND
75/19/18	Zanzibar	FAECES	17/04/2018	0.5	neg	ASSuAmcK
27/17/16	Enteritidis	FAECES	06/04/2016	0.25	neg	ND
57/3/16	Enteritidis	FAECES	13/05/2016	0.25	neg	Suscept
57/4/16	Enteritidis	FAECES	17/06/2016	0.25	neg	Suscept
57/7/16	Enteritidis	FAECES	19/06/2016	0.25	neg	Suscept
57/18/16	Enteritidis	FAECES	24/07/2016	0.25	neg	Suscept
57/15/16	Enteritidis	FAECES	24/07/2016	0.25	neg	ND
57/16/16	Enteritidis	FAECES	24/07/2016	0.25	neg	ND
57/17/16	Enteritidis	FAECES	24/07/2016	0.25	neg	ND
57/25/16	Enteritidis	FAECES	08/08/2016	0.25	neg	Suscept
57/26/16	Enteritidis	FAECES	11/08/2016	0.25	neg	Suscept
55/7/16	Enteritidis	FAECES	22/08/2016	0.25	neg	ND

55/30/16	Enteritidis	FAECES	16/09/2016	0.25	neg	ND
55/42/16	Enteritidis	FAECES	24/09/2016	0.25	neg	ND
27/23/16	Bovismorbificans	FAECES	23/03/2016	0.25	neg	Su
55/3/16	Coeln	FAECES	05/08/2016	0.25	neg	ND
57/8/16	Javiana	FAECES	28/06/2016	0.25	neg	Suscept
52/1/16	Napoli	FAECES	28/06/2016	0.25	neg	Suscept
55/25/16	Napoli	FAECES	20/08/2016	0.25	neg	ND
55/1/16	Newport	FAECES	15/07/2016	0.25	neg	ND
55/24/16	Paratyphi b	FAECES	07/09/2016	0.25	neg	Suscept
55/4/16	Rissen	FAECES	08/08/2016	0.25	neg	ND
55/21/16	Typhimurium	FAECES	02/08/2016	0.25	neg	ND
8/20/18	43:z4,z23:-	FAECES	30/10/2017	0.25	neg	SSu
75/1/18	Napoli	FAECES	20/06/2017	0.25	neg	Suscept
75/4/18	Napoli	FAECES	19/07/2017	0.25	neg	Suscept
75/5/18	Napoli	FAECES	08/08/2017	0.25	neg	Suscept
38/22/17	Typhi	FAECES	14/04/2017	0.25	neg	ND
75/2/18	Typhi	URINE	21/06/2017	0.25	neg	SNaPef
91/16/18	Enteritidis	BLOOD	24/08/2018	0.25	neg	Su
13/19/19	1,4,[5],12:i:-	FAECES	29/01/2018	0.25	neg	ASSuTTmpSxtNaPef
79/1/18	1,4,[5],12:i:-	FAECES	13/09/2018	0.25	neg	ASSuTNaPef
18/2/19	1,4,[5],12:i:-	FAECES	12/11/2018	0.25	neg	ASSuT
15/4/19	1,4,[5],12:i:-	FAECES	18/11/2018	0.25	neg	ASSuT
4/12/19	Goldcoast	FAECES	11/07/2018	0.25	neg	NaPef
4/27/19	Infantis	FAECES	17/03/2018	0.25	neg	ASSuTTmpSxtCazCtxNaPef
4/30/19	Infantis	FAECES	31/12/2018	0.25	neg	ASSuTTmpSxtCazCtxNaPef
4/31/19	Rissen	FAECES	23/11/2018	0.25	neg	ACSSuTTmpSxtKGNaPef

UNK: unknown information; ND: not determined; neg: negative PCR for *mcr-1* to *mcr-10* genes; 1,4,[5],12:i:- : *S*. Typhimurium monophasic variant; *: strain out of the surveillance period 2016-2018; suscept: Susceptible to all the other antimicobials tested; Na: nalidixic acid; Pef: pefloxacin; A: ampicillin; Ctx: cefotaxime; Caz: ceftazidime, Amc: amoxicillin/clavulanic acid 2:1; Fox: cefoxitin, C: chloramphenicol; G: gentamicin; K: kanamycin; S: streptomycin; Su: sulfonamides; T: tetracycline; Tmp: trimethoprim; Sxt: trimethoprim–sulfamethoxazole.
