# Supplementary material for: Colistin Resistance Mechanisms in Human Salmonella enterica Strains Isolated by the National Surveillance Enter-Net Italia (2016–2018)
Source: Antibiotics (Basel). 2022 Jan 13;11(1):102. doi: 10.3390/antibiotics11010102 (PMC8772777; doi:10.3390/antibiotics11010102)
Supplement: Supplementary file 1 [file antibiotics-11-00102-s001.zip › Supplementary Table S2.pdf]

Supplementary Table S2. Deleterious mutated proteins present in COL-R *mcr*-negative *S.* Enteritidis strains that were not further discussed in the study.

Protein name	PROVEAN	58/10/16	83/46/17	45/7/18
2-methylcitrate synthase, McsA	-6.946	G24D	no mutation	no mutation
Periplasmic alpha-amylase, MalS	-2.558	A538E	no mutation	no mutation
Cell division protein, FtsQ	-3.689	E141G	no mutation	no mutation
Protein translocase subunit, SecD	-3.625	P403L	no mutation	no mutation
Putative dihydroxyacid dehydratase, DHAD	-7.941	G185R	no mutation	no mutation
Propanediol dehydratase reactivation factor large subunit, PduG	-7.944	P403Q	no mutation	no mutation
Aspartokinase / Homoserine dehydrogenase, ThrA	-3.578	A215T	no mutation	no mutation
(lipoprotein)-autotransporter outer membrane beta-barrel domain-containing protein, YhjY	-7.992	R148W	no mutation	no mutation
Glucitol operon repressor, SrlR	-3.623	no mutation	no mutation	T31A
Pyrimidine / purine nucleotide 5'-monophosphate nucleosidase, PpnN	-6.555	no mutation	no mutation	D419A
Cobalt-precorrin-4 C (11)-methyltransferase, CbiF	-3.277	no mutation	no mutation	R225C
Transcriptional regulator, SlmA	-5.589	no mutation	no mutation	R31G
Enoyl-CoA hydratase / 3-hydroxyacyl-CoA dehydrogenase / 3-hydroxybutyryl-CoA epimerase, FadJ	-6.948	no mutation	no mutation	V326G
L-ribulose-5-phosphate 4-epimerase, AraD	-5.795	no mutation	no mutation	T117P
Flagellar motor switch protein, FliN	-5.741	no mutation	no mutation	Q83P
Lysine/cadaverine antiporter membrane protein, CadB	-2.561	no mutation	no mutation	L417Q
Argininosuccinate lyase, ArgH	-3.861	no mutation	no mutation	T155S
Propanediol dehydratase large subunit, PduC	-10.869	no mutation	no mutation	S264-E265insGS
3-oxo-tetronate kinase, OtnK (YgbK)	-2.841	no mutation	A91D	no mutation

no mutation: no mutation was detected in the protein sequence compared with the COL-S *S.* Enteritidis 4/23/16 and 56/1/16 strains.
