# Supplementary material for: Colistin Resistance Mechanisms in Human Salmonella enterica Strains Isolated by the National Surveillance Enter-Net Italia (2016–2018)
Source: Antibiotics (Basel). 2022 Jan 13;11(1):102. doi: 10.3390/antibiotics11010102 (PMC8772777; doi:10.3390/antibiotics11010102)
Supplement: Supplementary file 1 [file antibiotics-11-00102-s001.zip › Supplementary Table S3.pdf]

Supplementary Table S3: Analysis of *pilN*, *ydeI*, *lolB,* and *zraR* allele frequency in COL-R *mcr*-negative *S.* Enteritidis strains against wild type alleles, in the cgMLST database (pubMLST site).

**Gene (Locus name cgMLST)**	**N° of allele variants of cgMLST locus**	**N° of records in pubMLST**	**Strain with allele variant**
pilN (STMMW_34811)	493	31	58/10/16
	25	3,882	45/7/18, 83/46/17, 56/1/16, 4/23/16
ydeI (STMMW_15151)	302	4	58/10/16
	26	4,196	45/7/18, 83/46/17, 56/1/16, 4/23/16
lolB (STMMW_17701)	341	46	58/10/16
	20	4,403	45/7/18, 83/46/17, 56/1/16, 4/23/16
zraR (STMMW_41271)	1573	0	45/7/18
	29	3,962	58/10/16, 83/46/17, 56/1/16, 4/23/16
